# White Light-Emitting Diodes Based on AgInS_2_/ZnS Quantum Dots with Improved Bandwidth in Visible Light Communication

**DOI:** 10.3390/nano6010013

**Published:** 2016-01-08

**Authors:** Cheng Ruan, Yu Zhang, Min Lu, Changyin Ji, Chun Sun, Xiongbin Chen, Hongda Chen, Vicki L. Colvin, William W. Yu

**Affiliations:** 1 State Key Laboratory on Integrated Optoelectronics and College of Electronic Science and Engineering, Jilin University, Changchun 130012, China; rc_yinuo@163.com (C.R.); lumin15@mails.jlu.edu.cn (M.L.); jcy_168@126.com (C.J.); sunspring.518@163.com (C.S.); 2 State Key Laboratory of Integrated Optoelectronics, Institute of Semiconductors, Chinese Academy of Sciences, Beijing 100083, China; chenxiongbin@semi.ac.cn (X.C.); hdchen@semi.ac.cn (H.C.); 3 Department of Chemistry, Brown University, Providence, RI 02912, USA; colvin@rice.edu; 4 Department of Chemistry and Physics, Louisiana State University, Shreveport, LA 71115, USA

**Keywords:** white light-emitting diode (WLED), AgInS_2_/ZnS, quantum dot, color stability, modulation bandwidth, visible light communication

## Abstract

Quantum dot white light-emitting diodes (QD-WLEDs) were fabricated from green- and red-emitting AgInS_2_/ZnS core/shell QDs coated on GaN LEDs. Their electroluminescence (EL) spectra were measured at different currents, ranging from 50 mA to 400 mA, and showed good color stability. The modulation bandwidth of previously prepared QD-WLEDs was confirmed to be much wider than that of YAG:Ce phosphor-based WLEDs. These results indicate that the AgInS_2_/ZnS core/shell QDs are good color-converting materials for WLEDs and they are capable in visible light communication (VLC).

## 1. Introduction

With the increasing concerns in global climate change and environmental protection, people are looking for alternatives to reduce energy consumption and greenhouse gas emission [1,2]. Solid-state white light emitting diodes (WLEDs) are excellent candidates to replace conventional light sources because of their low power consumption, fast response, high luminous efficiency, excellent stability, and environmentally friendly characteristics [2,3]. The phosphors play an important role to fabricate WLEDs with high qualities [4,5]. Currently, the rare-earth phosphors represented by YAG:Ce^3+^ are prevalent in fabricating commercial WLEDs [6,7]. However, their color rendering is poor due to the lacking of red emission in the visible spectrum [2,8]. Quantum dots (QDs) have the size-dependent bandgap and high quantum yield, and have been widely applied in fabricating WLED. Based on the recent reports, CdSe QDs have been confirmed as a good down conversion materials for WLEDs [9]. However, the heavy-metal nature of the cadmium composition raises concerns about carcinogenicity and other chronic health risks as well as disposal hazards. Therefore, non-cadmium nanomaterials were proposed and prepared with the excellent optical properties, including CuInS_2_ QDs, AgInS_2_ QDs, InP/ZnS QDs and Silicon QDs [10,11,12,13,14,15], which have already been employed to fabricate the WLED and have demonstrated a promising application in the display and solid state lighting [16,17,18,19].

However, the inherent toxicity of cadmium-related QDs will limit their applications in commercial WLEDs and other related fields [20,21,22,23,24,25,26]. AgInS_2_/ZnS core/shell QDs were therefore investigated as desirable nontoxic substitutes [27,28,29]. Their photoluminescence (PL) wavelength can be adjusted from 520 nm to 680 nm with large Stokes shifts [30,31]. These characteristics enable AgInS_2_ QDs as color-converting materials to fabricate WLEDs.

In this work, we combined blue LED chips with green- and red-emitting AgInS_2_/ZnS QDs to construct WLEDs (QD-WLEDs). The PL lifetime of AgInS_2_ QDs was much shorter than that of the YAG:Ce^3+^ phosphor [32,33], and the corresponding modulation bandwidth was wider, making them ideal for improving system communication performance in visible light communication (VLC).

## 2. Experimental Section

### 2.1. Materials

Silver nitrate (AgNO_3_, 99%), indium (III) acetylacetonate (In(acac)_3_, 99.99%), sulfur powder (S, 99.98%), zinc stearate (10%–12% Zn basis), dodecanethiol (DDT, 98%), oleic acid (OA, 90%), oleylamine (OLA, 70%), 1-octadecene (ODE, 90%), and trioctylphosphine (TOP, 90%) were purchased from Sigma-Aldrich (Shanghai, China). UV glue NOA60 was ordered from LIENHE Fiber Optics (Shanghai, China). All chemicals were used directly without further purification. Blue LED chips (λ_max_ = 450 nm) and YAG:Ce-based WLEDs were purchased from the Cree company (Shenzhen, China).

### 2.2. Synthesis of AgInS_2_/ZnS Core/Shell QDs

AgInS_2_ core QDs were synthesized in a procedure performed previously [34]. A mixture of AgNO_3_ (0.1 mmol), In(acac)_3_ (0.5 mmol) and OA (1.5 mmol, 0.47 mL) were mixed and added into a 100 mL three-neck flask with ODE (25 mmol, 8.0 mL). The reaction mixture was degassed with N_2_ for 30 min at a room temperature. The solution was heated to 90 °C and DDT (4.0 mmol, 1.0 mL) was injected into the reaction flask. The mixture was then heated to 130 °C. The sulfur solution (0.80 mmol S powder dissolved in 1.3 mL OLA (3.1 mol/L)) was quickly injected into the reaction solution, and the solution continued reacting for 1–12 min. Different emission wavelength AgInS_2_ core QDs were obtained by changing the reaction time.

For the ZnS shell coating, a ZnS shell stock solution was prepared. Both Zn stearate (0.4 mmol) and S (0.4 mmol) were dissolved in TOP (4 mmol, 2 mL) and added to a 25 mL three-neck flask. The solution was degassed for 20 min and heated to 100 °C under a nitrogen flow until a clear colorless solution was formed. This solution was then quickly injected into the AgInS_2_ core solution at 130 °C. The temperature was maintained for 2 h. After that, the final solution was purified by adding anhydrous ethanol in order to remove the unreacted precursors, and this washing process was repeated three times [35,36,37]. The final core/shell QDs were dispersed in hexane.

### 2.3. Fabrication of WLEDs with AgInS_2_/ZnS Core/Shell QDs

A blue LED chip was used to generate blue light (450 nm) as a pump source. Green- and red-emitting AgInS_2_/ZnS core/shell QDs were dissolved in hexane, and UV glue was then added dropwise into each of the QD solutions. The treatments of vibration and sonication were applied for 30 min to form homogeneous mixtures. The two mixtures were then put in a vacuum chamber (2 × 10^3^ Pa) to remove hexane and bubbles. Finally, the two QD/UV glue mixtures were respectively dropped onto the blue LED chips layer by layer, and each layer was baked for 1 min under 365 nm ultraviolet light irradiation to harden the liquid on the LED chips.

### 2.4. Characterizations

Photoluminescence spectra were measured by a Zolix Omni-λ300 luminescence spectrometer (Zolix, Beijing, China). The UV-vis absorption spectra were obtained using a Shimadzu TU-1810 spectrophotometer (Shimadzu, Kyoto, Japan). Time-resolved photoluminescence spectra were measured by a fluorescence spectrometer (mini-τ, Edinburgh Photonics (Edinburgh Instruments Ltd., Edinburgh, UK) equipped with an EPL405 laser diode. When the decay curve was measured, a 5-μs separation was employed to avoid the PL accumulation. The modulation bandwidths of WLEDs were acquired using an Agilent 8714E network analyzer (300 KHz–3 GHz) (Agilent Technologies Inc., Santa Clara, CA, USA). A photoelectric detector BPW21 (Siemens Semiconductor Group, Shenzhen, China) was employed to switch the optical signal into an electrical signal. The absolute PL quantum yields were measured by the same spectrometer with an integrating sphere, with its inner face coated with BENFLEC^®^.

## 3. Results and Discussion

**Figure 1:**
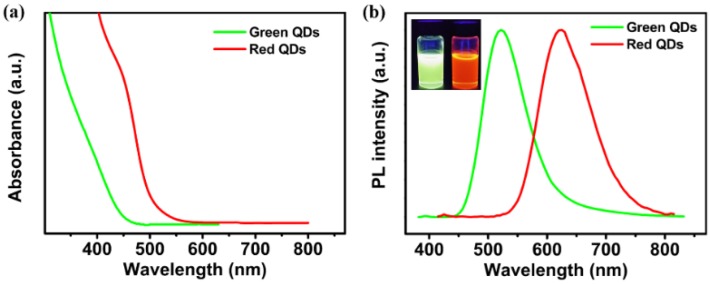
(**a**) Absorption spectra and (**b**) photoluminescence (PL) spectra of green- and red-emitting AgInS_2_/ZnS Quantum dots (QDs) in hexane. The inset shows the corresponding real color under excitation of 365 nm UV light.

The UV-vis absorption and PL spectra of green- and red-emitting AgInS_2_/ZnS QDs in hexane are depicted in Figure 1a,b. The PL peaks of AgInS_2_/ZnS QDs were 522 and 610 nm with the full width at half-maximum (FWHM) of 82 and 102 nm, respectively. The large Stokes shift was advantageous because it eliminated the self-absorption and generated high-color rendering WLEDs. The PL QYs of green- and red-emitting AgInS_2_/ZnS QDs were 50% and 40%, respectively. Under the excitation of 365 nm UV light, the strong green and red light could be clearly observed as shown in the inset of Figure 1b.

**Figure 2:**
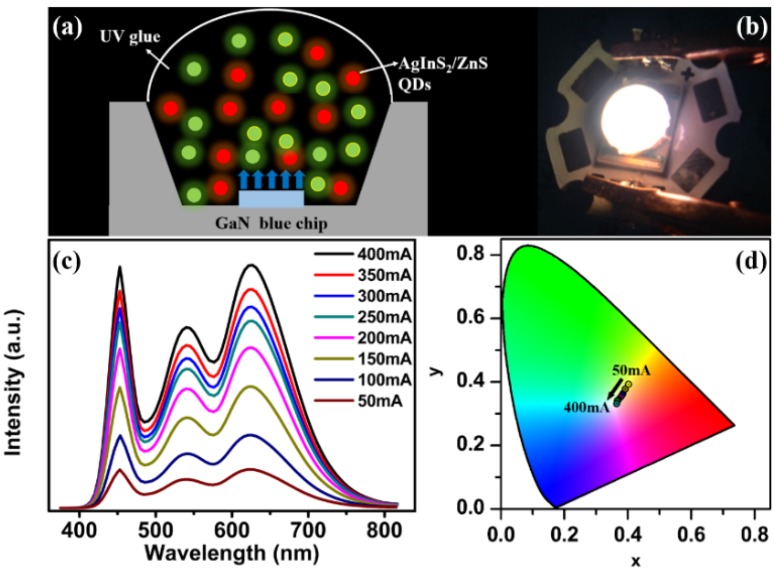
(**a**) The device structure for generating white light from green- and red-emitting AgInS_2_/ZnS QDs; (**b**) the real emitting color picture operated at 350 mA; (**c**) electroluminescence (EL spectra) of AgInS_2_ Quantum dot white light-emitting diode (QD-WLED) at different working currents from 50 mA to 400 mA; (**d**) the corresponding CIE coordinates of the QD-WLED.

Figure 2a shows the schematic structure of the QD-WLEDs with green- and red-emitting AgInS_2_/ZnS QDs. A photograph of the WLED operated at a working current of 350 mA is shown in Figure 2b. Compared to the poor CRI of the YAG:Ce-based WLED, the AgInS_2_/ZnS QD-WLED has the improved CRI of 85. Figure 2c shows the electroluminescence (EL) spectra of the as-fabricated QD-WLED under different currents from 50 mA to 400 mA. Three emission peaks were clearly located at 450 nm, 540 nm, and 625 nm, respectively. It was noted that a redshift about 20 nm was observed for the QD peak wavelengths of QD-WLED compared to their PL spectra. Because of the low QD solubility in the UV glue, QD agglomeration occurred inevitably. The dipole-dipole energy transfer, which was strongly dependent on QD distance, became enhanced and led to an obvious redshift due to the energy transfer between QDs. In addition, it is known that the exciton binding energy of QDs can be affected by the dielectric constant of the surrounding media. Due to the difference in the dielectric constants of the QD-surrounding dispersion media (hexane for PL *versus* UV glue for EL), the redshift can also happen [38]. Figure 2d shows the Commission International de I’Eclairage (CIE) coordinates of the QD-WLED operated at different currents from 50 mA to 400 mA, which were each calculated through the EL spectra.

**Figure 3:**
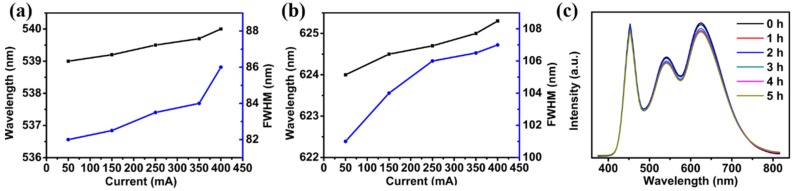
The peak positions and the full width at half-maximum (FWHM) of QDs in the white light emitting diode (WLED) under different currents from 50 mA to 400 mA for (**a**) green- and (**b**) red-emitting AgInS_2_/ZnS QDs; (**c**) PL spectra of the WLED at different working time when the working current was 350 mA.

Figure 3 shows the output spectra (EL) peaks and the corresponding FWHMs of green- and red-emitting AgInS_2_/ZnS QDs coated on a blue LED chip as a function of the applied current (original data from Figure 2c). The blue line shows the FWHMs of the WLED emissions broadened from 82 to 86 nm for green-emitting QDs (Figure 3a) and from 101 to 107 nm for red-emitting QDs (Figure 3b) under the different currents, respectively. The EL peaks of green- and red-emitting AgInS_2_/ZnS QDs, which were excited by blue LED, were 539 nm and 624 nm at 50 mA as shown in Figure 3 (black line), respectively. As the current increased, the peak position appeared slightly redshifted. When the current increased from 50 mA to 400 mA, the spectra of two QDs shifted from 539 to 540.3 nm and 624 to 625.7 nm, respectively. The peak positions showed small variations of only 1.3 nm and 1.7 nm, respectively. This result indicated that the variations of the EL spectra and the FWHM for QD-WLEDs were very small in spite of the large changes in current, which means that the AgInS_2_/ZnS QD-WLEDs were quite stable under the typical LED operating currents. Figure 3c exhibits the PL spectra of the WLED at different working time. The PL intensity of AgInS_2_/ZnS QDs decreased slightly, which was mainly due to the increased temperature on the surface of blue chips.

**Figure 4:**
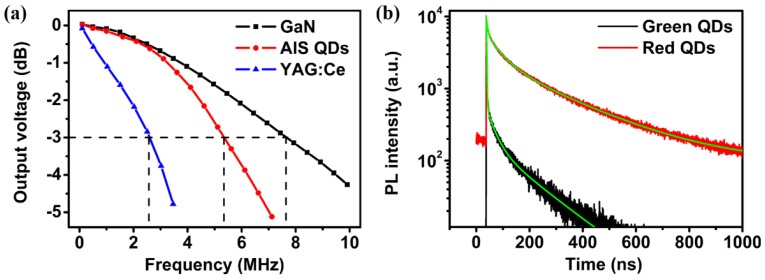
(**a**) The frequency responses of blue GaN LED (black line), AgInS_2_/ZnS QD-WLED (red line), and YAG:Ce WLED (blue line) at 350 mA, respectively; (**b**) PL decay curves of green- and red-emitting AgInS_2_/ZnS QDs.

The modulation bandwidth of a WLED depends on the bandwidth of pumping blue GaN LED. However, the phosphors on the surface of blue LED also had a great influence on the modulation bandwidth. As shown in Figure 4a, the converted output voltage intensity of the blue LED, the as-fabricated QD-WLED and the commercial YAG:Ce WLED were measured as the frequency increased from 100 Hz to 10 MHz. It can be seen that the modulation bandwidths were 2.59 MHz for the commercial YAG:Ce WLED and 7.65 MHz for the blue LED, because the long decay time of the YAG:Ce phosphor limited the available bandwidth.

The PL mechanisms of YAG:Ce phosphors and QDs are different. The radiative relaxation of YAG:Ce is related to the trap energy state with certain depth, which causes the long afterglow with the lifetime of a few microseconds [39]. Donor-acceptor (D–A) pair recombination and/or near-bandedge emission have been proposed to explain the PL mechanism of AgInS_2_ or CuInS_2_ QDs with the PL lifetime of typically a few hundred nanoseconds [34,36]. This emission lifetime was longer than that of band-edge emission materials [40], and the large “global” Stokes shift between the PL band and the band-edge absorption feature indicate that the radiative recombination in these NCs involves a localized intragap state, which can be an internal defect. Therefore, this PL decay time demonstrates the existence of defect-related recombination in the QDs, including the donor-acceptor (D–A) pair recombination and/or near-band-edge recombination.

Figure 4b shows the PL decay curves of our green- and red-emitting AgInS_2_/ZnS QDs with the PL lifetimes of 77 ns and 193 ns, respectively, which was shorter than that of YAG:Ce [34,35,36,37,38,39,41]. Obviously, the PL lifetimes of AgInS_2_/ZnS QDs were much shorter than that of the yellow YAG:Ce phosphor [32,33]. The modulation bandwidth of AgInS_2_/ZnS QD-WLED was measured as approximately 5.4 MHz, which was much higher than the bandwidth of the commercial YAG:Ce WLED.

## 4. Conclusions

In summary, WLEDs were fabricated, combining green- and red-emitting AgInS_2_/ZnS QDs with blue GaN LEDs. The CRI was improved to 85 in comparison to the commercial YAG:Ce WLED, meaning that the AgInS_2_/ZnS QDs were suitable to fabricate WLEDs as down conversion materials. The EL spectra of QD-WLEDs showed a good stability. Finally, the modulation bandwidth of QD-WLEDs was greatly improved compared to that of YAG:Ce WLEDs. Therefore, these results suggest that AgInS_2_/ZnS QDs are promising phosphors, not only in terms of generating high quality white light, but also improving the bandwidth in visible light communication.
